# In Vitro Fertilization Outcomes With a Dual Trigger in Normoresponders in Antagonist Cycles

**DOI:** 10.7759/cureus.45623

**Published:** 2023-09-20

**Authors:** Munire Funda Cevher Akdulum, Seçil İrem Arık, Erhan Demirdağ, Mehmet Erdem, Ahmet Erdem

**Affiliations:** 1 Obstetrics and Gynaecology, Gazi University, Ankara, TUR; 2 Obstetrics and Gynecology, Gazi University, School of Medicine, Ankara, TUR; 3 Obstetrics and Gynecology, Gazi University, Ankara, TUR

**Keywords:** live birth rate, normoresponder, dual trigger, antagonist cycle, in vitro fertilization ivf

## Abstract

Objectives

To evaluate whether the dual trigger of ovulation with a gonadotropin-releasing hormone (GnRH) agonist and the standard dose of recombinant human chorionic gonadotropin (hCG) (dual trigger) is better than hCG alone in in vitro fertilization (IVF) cycles of patients who responded well to ovarian stimulation.

Methods

Between January 2013 and December 2021, 5593 antagonist cycles of patients were reviewed. This study included women who had an antral follicle count of 5 or more and exhibited a normoresponse to ovarian stimulation using the GnRH antagonist protocol, as determined by the follicular output rate (FORT). The primary outcome indicators consisted of the quantities of retrieved oocytes and mature oocytes. The secondary outcome markers included live birth rates, clinical pregnancy rates, and continued pregnancy rates.

Results

A total of 1244 normoresponder women who met the inclusion criteria were identified from the scanned files and subsequently enrolled in the GnRH antagonist protocol. A total of 383 cycles were observed in the group that was given the standard hCG trigger while 861 cycles were observed in the group that was given the dual trigger. The number of mature oocytes and top-quality embryos was significantly higher in the dual trigger group. The maturation rate in the hCG group was 74.8% while it was 76.9% in the dual trigger group (p=0.018). The dual trigger group exhibited an ongoing pregnancy rate of 37.6%, whereas the hCG group had a rate of 30.1% (p = 0.02). The dual trigger group exhibited a slightly higher live birth rate (34.3% vs 29.2%, p = 0.11), although this difference did not reach statistical significance.

Conclusion

Dual trigger of ovulation was superior to hCG alone in terms of the number of mature oocytes yielded, top quality of embryos, maturation rates, and ongoing pregnancy in IVF cycles of normoresponders having ovarian stimulation on the GnRH antagonist protocol.

## Introduction

Human chorionic gonadotropin (hCG) has long been used in the final stages of oocyte maturation and has been found to increase pregnancy rates. However, due to the long half-life of hCG, injection of hCG for final oocyte development results in higher-than-normal steroid levels in the luteal phase, which is associated with an increased risk of ovarian hyperstimulation syndrome (OHSS) [1]. According to some studies, the hCG trigger may have a potentially negative effect on the quality of the embryo and endometrium [2].

hCG has been supplemented with gonadotropin-releasing hormone (GnRH) agonists in GnRH antagonist regimens during in vitro fertilization (IVF) cycles to stimulate ovulation [3]. The advantages of GnRH agonist (GnRHa) stimulation include reducing the risk of OHSS and increasing the number of mature oocytes by causing an increase in both FSH and LH [4-8]. Previously, the main disadvantage of a GnRHa trigger was the reduction of LH and the stimulus for the corpus luteum of LH loss; this resulted in a lower live birth rate and a higher risk of miscarriage [9]. However, luteal phase hCG injection and substantial luteal phase support have solved this problem [10].

Few studies have looked at the effects of dual triggering on IVF outcomes in different subgroups of women such as hyper-responders, normoresponders, and poor responders. Even fewer studies report live birth rates, the ultimate goal of infertility treatment, in women with numerous triggers and predicted normal ovarian response [2,11-15].

In order to improve IVF results in normoresponders, we set out to research the possible benefits of the dual trigger.

## Materials and methods

A retrospective cohort study was conducted at the Novaart IVF Center in Ankara, a private infertility clinic, spanning from January 2013 to December 2021. The study received approval from the Ethical Committee of Gazi University (2022-952). The analysis encompassed a comprehensive dataset of 5593 instances of intracytoplasmic sperm injection utilizing antagonist medication, derived from the medical records of the clinic. The study excluded patients who had high or low body mass indexes (>30 kg/m^2^), as well as those who had additional endocrine disorders such as diabetes, thyroid dysfunction, hyperprolactinemia, congenital adrenal hyperplasia, and Addison's disease. Patients with uterine anomalies and women with a poor prognosis at the time of treatment initiation were also excluded from the study. Additionally, freeze-all cycles were not incorporated. The study encompassed instances of infertility that lacked a clear etiology. This study included women who had an antral follicle count of 5 or more and exhibited a normoresponse to ovarian stimulation using the GnRH antagonist protocol, as determined by the follicular output rate (FORT). In the study, only women with ovulatory cycles were included.

Both groups underwent exogenous gonadotropin stimulation, with doses of up to 400 units, using recombinant FSH (Gonal-F, Merck Serono, Turkey) combined with hMG (Menogon, Ferring, Turkey). In order to evaluate the ovarian response to gonadotropin stimulation, a series of transvaginal ultrasonography examinations, serum estradiol tests, monitoring of follicular development, and adjustments to the dosage of gonadotropins were performed. All sonographic procedures were performed utilizing the same device. The injection of subcutaneous cetrorelix (Cetrotide; Asta Medica, Frankfurt, Germany) at a dosage of 0.25 mg per day was initiated and maintained until the day of ovulation trigger, which was determined by the presence of a leading follicle measuring 13 mm or an estradiol level exceeding 300 pg/mL. In the study, the initiation of final oocyte development was achieved by administering 250 mcg of choriogonadotropin alfa (recombinant hCG) in the control group, or by administering 250 mcg of recombinant hCG (choriogonadotropin alfa) (Ovitrelle, Merck Serono, Turkey) along with 0.2 mg of triptorelin (Decapeptyl, Ferring, Turkey) in the study group. This was done when a minimum of two follicles, with a mean diameter of 8 mm, had increased by 16 mm.

The oocyte pick-up procedure was performed with the assistance of sonographic guidance, precisely 36 hours after the administration of the ovulation trigger. During the procedure of general anesthesia, the follicles were aspirated, and the metaphase II (MII) oocytes underwent intracytoplasmic sperm injection (ICSI) fertilization. The transfer of high-quality embryos was conducted by employing a flexible catheter under the guidance of ultrasonography, within a timeframe of three to five days after the retrieval of oocytes. This procedure was carried out using the Wallace catheter and Irvine Scientific's facilities located in Santa Ana, CA. The regulation of the luteal phase involved the administration of 90 mg of intravaginal progesterone gel daily (namely, Crinone 8% gel manufactured by Serono) and 4 mg of oral estradiol hemihydrate (specifically, Estrofem produced by Novo Nordisk, Turkey). This treatment commenced the day following oocyte pick-up and continued until the 10th week of gestation. The announcement of pregnancy occurred with the detection of a positive serum hCG level on the twelfth day following the transfer of the embryo. The term "clinical pregnancy" refers to the identification of a gestational sac or fetus exhibiting cardiac activity through the use of transvaginal ultrasound imaging. A live birth was defined as a viable fetus born at or after 22 weeks of gestation.

The primary outcome indicators consisted of the quantities of retrieved oocytes and mature oocytes. The secondary outcome markers included live birth rates, clinical pregnancy rates, and continued pregnancy rates.

All statistical analyses were conducted using the Statistical Package for Social Sciences (SPSS, version 21.0, Statistics, 2013, Chicago, IBM, USA). The data from a normal distribution has been evaluated for normality by statistical methods such as the Kolmogorov-Smirnov test. The statistical analysis employed in this study involved the utilization of the student's t-test to compare parametric data that had a consistent distribution. The Mann-Whitney U test was employed to assess the differences between datasets that did not follow a normal distribution. The chi-square test or Fischer's exact test was employed to examine discrepancies within categorical data. The mean and standard deviation were used to present the continuous variables. The presentation of categorical data involved the representation of percentages, whereas data exhibiting a non-normal distribution were depicted using the median. The statistical significance level was set at a p-value of 0.05.

## Results

A total of 1244 normoresponder women who met the inclusion criteria were identified from the scanned files and subsequently enrolled in the GnRH antagonist protocol. A total of 383 cycles were observed in the group that was given the standard hCG trigger while 861 cycles were observed in the group that was given the dual trigger. Table 1 shows that the demographic characteristics of the groups, such as age, BMI, basal follicle-stimulating hormone (FSH), antral follicle count (AFC), and duration of infertility, were comparable.

**Table 1:** Comparison of baseline characteristics between groups Data were presented as mean ± SD. p <0.05 was considered significant. BMI: Body mass index; FSH: Follicle-stimulating hormone; AFC: Antral follicle count

Variables	hCG trigger group (n = 383 cycles)	Dual trigger group (n = 861 cycles)	p-value
Age (years)	33.1 ± 5.2	33.6 ± 5.1	0.09
BMI (kg/m^2^)	22.0 ± 1.5	21.9 ± 2.1	0.48
Basal FSH (mIU/ml)	6.5 ± 2.4	6.8 ± 2.5	0.53
AFC (n)	9.3 ± 2.1	9.1 ± 2.1	0.25
Duration of infertility (years)	6.0 ± 4.5	5.9 ± 4.2	0.07

Table 2 reveals the characteristics of the menstrual cycle and the outcomes of pregnancy. The concentrations of progesterone, estradiol, and luteinizing hormone (LH) on the day of the trigger were comparable in both groups. In the hCG group, there was a notable increase in both the duration of stimulation and the total dose of administered gonadotropin. There was no significant difference observed in the number of follicles measuring greater than 17 mm on the day of hCG injection and the total number of oocytes retrieved between the groups. The study group, which utilized the dual trigger group method, exhibited a notably greater quantity of mature oocytes (7.2 ± 2.9 vs 6.7 ± 2.6, p=0.02) and top-quality embryos (2.2 ± 1.7 vs 1.5 ± 1.3, p=0.00) that were transferred, in comparison to the control group. The dual trigger group exhibited a slightly higher mean number of retrieved oocytes (9.3 ± 3.0 vs 9.0 ± 2.9, p=0.11). The maturation rate in the hCG group was 74.8% while it was 76.9% in the dual trigger group. The observed discrepancy exhibited statistical significance. The statistical significance level of the test was found to be p=0.018. The rates of fertilization exhibited no significant differences between the groups. The dual trigger group exhibited an ongoing pregnancy rate of 37.6%, whereas the hCG group had a rate of 30.1% (p = 0.02). The dual trigger group exhibited a slightly higher live birth rate (34.3% vs 29.2%, p = 0.11), although this difference did not reach statistical significance.

**Table 2:** Comparison of cycle characteristics and pregnancy outcomes between groups Data were presented as mean ± SD, median (25-75 percentile), numbers, and percentages. p <0.05 was considered significant. E2: Estradiol; P: Progesterone;LH: Luteinizing hormone, hCG: Human chorionic gonadotropin, MII: Metaphase 2

Variables	hCG trigger group (n=383)	Dual trigger group (n=861)	p-value
Duration of stimulation (days)	10.7 ± 2.0	10.4 ± 1.6	0.02
Total dose of gonadotropins (IU)	3046 ± 821	2880 ± 642	0.00
Serum E2 on the day of hCG injection (pg/ml)	2716 ± 1746	2569 ± 1617	0.20
Serum P on the day of hCG injection (ng/ml)	0.9 ± 0.7	0.9 ± 0.7	0.97
Serum LH on the day of hCG injection (mIU/ml)	3.3 ± 3.0	3.0 ± 2.0	0.10
Number of follicles > 17mm on the day of hCG injection (n)	11.0 ± 2.9	11.3 ± 3.0	0.11
Number of total oocytes retrieved	9.0 ± 2.9	9.3 ± 3.0	0.11
Number of mature oocytes (MII)	6.7 ± 2.6	7.2 ± 2.9	0.02
Top-quality embryos	1.5 ± 1.3	2.2 ± 1.7	0.00
Ongoing pregnancy rate, per cycle (%)	(30.1%)	(37.6%)	0.02
Live birth rate, per cycle, n (%)	99 (29.2%)	221 (34.3%)	0.11
Maturation rates (%)	74.8	76.9	0.018
Fertilization rates (%)	78.1	79.4	0.12

## Discussion

The study was conducted at a single institution, focusing on a cohort of individuals classified as normal ovarian responders. The findings of our study indicate that the group subjected to dual trigger exhibited a statistically significant increase in the number of mature oocytes, top-quality embryos, ongoing pregnancy rates, and maturation rates. There was no statistically significant disparity observed in the rates of live birth and fertilization.

The findings of Ali et al. from a randomized controlled study involving a smaller sample size provide support for our findings. The study found that women in the dual trigger group exhibited a significantly higher number of retrieved oocytes (p = 0.001), MII oocytes (p = 0.01), and grade one embryos (p = 0.04) compared to other groups [13]. The results of our study align with the findings reported by Lin et al. and Schachter et al. The studies demonstrated a significant increase in the number of retrieved oocytes and MII oocytes [12,15].

In a double-blind, randomized controlled study conducted by Haas et al. in 2020 [16], it was observed that the dual trigger group exhibited a greater quantity of oocytes, mature oocytes, blastocysts, top quality blastocysts, implantation rate, continued pregnancy rate, and live birth rate. While the logistic regression analysis did not provide support for the comparison of live birth rates in this study, it was observed that the group receiving the dual trigger had a higher number of high-quality embryos and a greater number of cumulative pregnancies [16]. There was no statistically significant disparity observed in the live birth rates between the study conducted by Haas et al. and our own study. However, it is worth noting that the dual trigger group exhibited a comparatively higher proportion of live births. In a recent study conducted by Gao et al., it was observed that there existed a notable disparity in the quantity of oocytes obtained [17]. However, no noticeable difference was observed in the number of high-quality embryos or the cumulative rates of pregnancy.

The dual trigger effect may have potentially expedited the process of oocyte maturation or exerted a direct influence on implantation. Endometrial cells that have activated GnRH receptors regulate mitotic cell division and differentiation [18,19], as well as extracellular matrix degradation and trophoblastic invasion, by producing insulin-like growth factor, epidermal growth factor, and mitogen-activated protein kinase [20,21]. Gonadotropin-releasing hormone serves as a physiological ovulation trigger by inducing both the LH and FSH surges during the mid-cycle. This dual action of GnRH provides an additional benefit. The augmentation of LH activity, through the administration of GnRH analogs in conjunction with the hCG trigger, has the potential to address various challenges related to the endocrine domain such as granulosa cell dysfunction, impaired oocyte maturation, and insufficient cumulus development [22,23].

A systematic review and meta-analysis was conducted, encompassing a total of four studies and involving a sample size of 527 patients. The study conducted by Chen found that there was a notable increase in clinical pregnancy rates. However, there was no statistically significant difference observed in ongoing pregnancy rates [24]. A subsequent meta-analysis, encompassing a sample size of 1048 patients, revealed statistically significant increases in the quantities of collected oocytes, fertilized oocytes, clinical pregnancy rates, and live birth rates [25]. In our study, there was a notable increase in the quantity of mature oocytes and high-quality embryos. According to a recent meta-analysis conducted by Gonzalez, it was found that the dual trigger group exhibited significantly higher rates of retrieved oocytes and clinical pregnancy when compared to other groups [26].

Our study contributes to the existing body of literature because it was conducted on a total of 1244 individuals who were treated at a single center. The fact that our research was carried out in retrospect is one of the limitations of our study. There was no group in our research that was solely activated by triptorelin, which was another point of differentiation between the two studies. If we had included a third arm in the trial, we would have been able to determine, based on the results, whether the improved outcome was due to the administration of triptorelin alone or to the administration of triptorelin in conjunction with hCG.

## Conclusions

Consequently, we have reached the conclusion that the implementation of dual triggers in antagonist cycles resulted in an augmentation of both the quantity of mature oocytes and the quality of embryos in the normoresponder group. There was no statistically significant difference observed between the rates of live births and clinical pregnancy rates.
